# Runs of homozygosity and population history in cattle

**DOI:** 10.1186/1471-2156-13-70

**Published:** 2012-08-14

**Authors:** Deirdre C Purfield, Donagh P Berry, Sinead McParland, Daniel G Bradley

**Affiliations:** 1 Smurfit Institute of Genetics, University of Dublin, Trinity College, Dublin 2, Ireland; 2 Animal & Grassland Research and Innovation Center, Teagasc, Moorepark, Fermoy, Co, Cork, Ireland

**Keywords:** Runs of homozygosity, Inbreeding, Cattle population history

## Abstract

**Background:**

Runs of homozygosity (ROH) are contiguous lengths of homozygous genotypes that are present in an individual due to parents transmitting identical haplotypes to their offspring. The extent and frequency of ROHs may inform on the ancestry of an individual and its population. Here we use high density (n = 777,962) bi-allelic SNPs in a range of cattle breed samples to correlate ROH with the pedigree-based inbreeding coefficients and to validate subsequent analyses using 54,001 SNP genotypes. This study provides a first testing of the inference drawn from ROH through comparison with estimates of inbreeding from calculations based on the detailed pedigree data available for several breeds.

**Results:**

All animals genotyped on the HD panel displayed at least one ROH that was between 1–5 Mb in length with certain regions of the genome more likely to be involved in a ROH than others. Strong correlations (r = 0.75, p < 0.0001) existed between the pedigree-based inbreeding coefficient and a statistic based on sum of ROH of length > 0.5 KB and suggests that in the absence of an animal’s pedigree data, the extent of a genome under ROH may be used to infer aspects of recent population history even from relatively few samples.

**Conclusions:**

Our findings suggest that ROH are frequent across all breeds but differing patterns of ROH length and burden illustrate variations in breed origins and recent management.

## Background

Runs of homozygosity (ROH) are contiguous lengths of homozygous genotypes that are present in an animal due to parents transmitting identical haplotypes to their offspring. The extent and frequency of these may inform on the ancestry of an individual and its population. Particularly, consanguinity may be indicated from the presence of long ROH; the longer such segments are, the more likely that recent inbreeding occurred within a pedigree [1]. However, unusually long runs of homozygosity may also persist in out bred individuals, perhaps due to unusual mutation, linkage disequilibrium (LD), and recombination rates at certain genomic locations [2].

The distribution of shorter ROH may also inform on the presence of more ancient relatedness which is unaccounted for in an individual’s recorded pedigree due to the limitations in the recording process [3]. In the context of domestic animals these may result from breed or population founder effects or other restrictions.

The domestication of cattle, which occurred ~10,000 years ago, was a complex process, with evidence suggesting that it occurred in a minimum of two domestication events [4]. However, both natural and artificial selection of cattle, as well as regional variations due to drift has resulted in breeds that differ extensively in phenotypes. These processes and the extent of breeding control have differed greatly among populations and ROH may provide useful information on these disparate histories. Particularly, in recent times the practices of intense selection of sires, artificial insemination, and embryo transfer have featured heavily in some breeds, reducing effective population sizes, genetic diversity and affecting levels of homozygosity.

Runs of homozygosity have been extensively studied in human populations and are an established method of distinguishing a population history of consanguinity, and with homozygosity mapping analysis showing a relationship with susceptibility to recessive diseases [1-3,5,6]. Here we use high density (n = 777,962) bi-allelic SNP data in a range of cattle breeds to correlate ROH with the pedigree-based inbreeding coefficient and to validate further analysis using 54,001 SNP genotypes. This allows examination and interpretation of the level of ROH that exist in a wide range of cattle breeds samples.

## Methods

### Genotypes and quality control

Single nucleotide polymorphisms (SNPs) genotypes consisting of 777,972 bialleleic SNPs from the BovineHD BeadChip (Illumina Inc., San Diego, CA) were generated for 891 artificial insemination sires of multiple breeds. Breeds represented included Angus (n = 39), Belgian Blue (n = 38), Charolais (n = 117), Friesian (n = 98), Hereford (n = 40), Holstein (n = 262), Holstein-Friesian crosses (n = 111), Limousin (n = 128) and Simmental (n = 58). Additionally, the 48,734 SNPs common to both the HD and the Illumina BovineSNP50 Beadchip were retained in a reduced HD panel which was used to evaluate the lesser *circa* 50 k SNP density for identifying ROH; hereafter referred to as the reduced HD panel . In addition, three published genotype datasets at this density comprising of a total of 1166 animals from 42 different breeds (detailed in Additional file 1.) generated from the Illumina Bovine50 Beadchip, hereafter known as the SNP50 panel, were also available [7-9].

The impact of the following SNP edits on the number of SNPs remaining for each of the three density panels is summarised in Table 1. Only biallelic SNPs on the 29 autosomes were retained and both animals and SNPs with call rates <90% were discarded. Also, monomorphic SNPs and SNPs that deviated from the Hardy Weinberg equilibrium (p < 0.0001) within breed were discarded.

**Table 1:** Number of single nucleotide polymorphisms for each data edit implemented on each density panel

**Genotype edit**	**HD Panel**	**Reduced HD Panel**	**Hapmap SNP50**	**Gautier SNP50**
Initial data set	777,962 (891)	48,734 (891)	54008 (533)	58336 (633)
Autosomes only	735,293 (891)	47,270 (891)	51587 (533)	55200 (633)
SNP and animals with >90 % Call Rate	727,559 (867)	46,761 (867)	50824 (530)	44687 (633)
Hardy Weinburg Equilibrium in each breed (p < 0.0001)	723,502 (867)	46,379 (867)	50783 (530)	44602 (633)
Monomorphic SNPs removed	665,058 (867)	43,028 (867)	48128 (530)	44160 (633)

The SNP edits that were applied to each dataset detailing the amount of SNPs removed and the number of animals in each dataset is listed in parenthesis.

### Definition of a run of homozygosity

Runs of homozygosity (ROH) were defined in the populations of animals for each of the three SNP density panels using PLINK v1.07, which slides a window of 50 SNPs, in one SNP intervals, across the genome estimating homozygosity. No more than two SNPs with missing genotypes were allowed per window and up to one possible heterozygous genotype was permitted. To ensure low SNP density did not affect ROH length, the minimum required density of SNPs per kb differed between the two genotyping densities, as well as the maximum distance length between two consecutive homozygous SNPs in a run. To minimize the number of ROH that occur by chance in the HD panel, the minimum number of SNPs that constituted a ROH (*l*) was calculated by a method similar to that proposed by Lencz et al., [6].

$$l=\frac{\log_{e}\frac{a}{n_{s}.n_{i}}}{\log_{e}(1-\bar{het)}}$$

where n_s_ is the number of SNPs per individual, n_i_ is the number of individuals, α is the percentage of false positive ROH (set to 0.05 in the present study), $\bar{het}$ is the mean SNP heterozygosity across all SNPs. For the exclusion of very short and common ROH that occur prevalently throughout the genome due to LD, a minimum ROH length of 500 kb was set.

For analysis of the HD panel genotypes, the minimum SNP density was 1 SNP every 50 kb to ensure low SNP density did not falsify ROH length, a minimum run length of 58 SNPs was needed to produce <5% randomly generated ROH and the maximum gap between two consecutive homozygous SNPs in a run was set at a 100 kb. In the analysis of the reduced HD panel and the Bovine SNP50 panel, the minimum SNP density was altered to 1 SNP every 120 kb and no restriction was placed on the minimum number of SNPs in a ROH and the maximum gap length between two consecutive homozygous SNPs in a run was kept at the default value of 1000 kb to account for the lesser genotype density.

Runs of homozygosity were identified for each animal separately on the HD and reduced panel and for comparative purposes only, the maximum gap length in the HD panel for ROH identification was altered to 1000 kb, SNP density to 1 SNP every 120 kb and there was no restriction on the minimum number of SNPs that constituted a ROH, in order to limit bias. To establish that the reduced density also predicts the correct ROH length category for ROH, the extent that reduced panel ROH were correctly assigned to the HD ROH length category was plotted.

Animals with overlapping ROH, and those ROH that were an allelic match, were also identified in the HD panel. The identification of overlapping regions was done by using the sliding window approach as mentioned above, and then for each SNP by calculating the proportion of homozygous windows in the population dataset that overlap that same position. The percentage of animals that had the region with the most overlapping ROH on each chromosome was plotted and the percentage of these overlapping ROH that were an allelic match ≥ 95 % were identified.

The percentage population of each breed with ROH present at different ROH length categories was calculated, as well as the mean overall ROH sum per animal for each breed. The mean sum of ROH within each ROH length category was also calculated by summing all ROH per animal in each ROH length category and averaging this per breed population. The percentage of SNP involvement in ROH was also calculated by counting the amount of times a SNP appeared in a ROH in the population dataset.

### Inbreeding coefficient vs. runs of homozygosity

The measure of homozygosity per animal was calculated using the HD panel and reduced HD panel, by a method similar to that as proposed by McQuillan et al., (2008) except in the present study the centromeric region was also included in the calculation,

$$F_{\mathrm{ROH}}=\sum \frac{L_{\mathrm{ROH}}}{L_{\mathrm{AUTO}}}$$

in which L_ROH_ is the sum of ROH per animal above a certain criterion length and L_AUTO_ is the total length of autosome covered by SNPs. L_ROH_ was calculated separately as the sum of all ROH >500 kb and the sum of all ROH >10,000 kb where SNP autosomal genome coverage was 2,510,611 kb in the HD panel and 2,500,265 kb in the reduced HD panel. Pedigree based inbreeding coefficients for all animals were calculated using the Meuwissen and Luo [10] algorithm. Depth of pedigree known was measured in complete generation equivalents (CGE) for all animals as described in [11] and correlations between all measures of inbreeding were calculated only on animals (n = 230) with a CGE value ≥6.

## Results

### ROH in animals with HD panel genotypes

**Figure 1:**
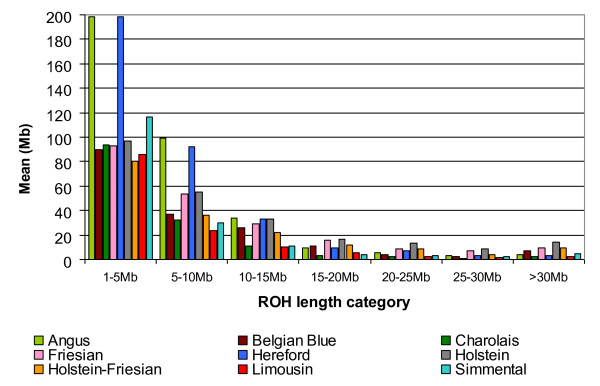
**The mean sum of Run of Homozygosity (ROH) per HD genotyped animal.** The sum of ROH was calculated per animal, measured in megabases (Mb) within each ROH length category and averaged per breed. Breeds from left to right are Angus, Belgian Blue, Charolais, Friesian, Hereford, Holstein, Holstein-Friesian, Limousin and Simmental. Angus and Hereford had high amount of mean short ROH (<5 Mb) possibly due to ancestral relatedness whereas the Holsteins had the greater mean long ROH (>20 Mb) due to recent consanguineous matings.

We used a definition of ROH as tracts of homozygous genotypes that were >500 kb in length identified in a genome sliding window of 50 SNPs. No more than two missing genotypes and one possible heterozygous genotype were allowed in a window, and within our HD SNP panel data, all 867 animals tested, displayed at least one ROH that was between 1–5 Mb in length with almost all (i.e., 98%) of the population dataset also having at least one ROH between 5–10 Mb in length. Differences among breeds existed in their frequency in different ROH length categories (Figure 1). The two British breeds, the Angus and the Hereford, had a larger mean portion of their genome, 198.6 Mb and 198.7 Mb, respectively, covered in shorter length (i.e. 1–5 Mb) ROH; coverage ranged from 80.58 to 93.48 Mb in the remaining breeds. For all breeds, most ROH segment coverage was in the shorter length categories with the Holstein, Holstein-Friesian and Friesian samples showing the greatest frequency in the longer ROH categories which are more indicative of recent inbreeding. A smaller effective population size and its associated unfavourable effects due to inbreeding depression in these dairy cattle is a current concern in the industry [12]. The mean sum of the ROH per animal for all ROH length categories > 15-20 Mb was less than 20 Mb and no animal had more than five ROH that were >30 Mb in length. The three most homozygous animals present in our dataset were from the dairy breeds and had on average 700.3 Mb classified as ROH; this is equivalent to almost a quarter of their genome.

### Genome locations of ROH

**Figure 2:**
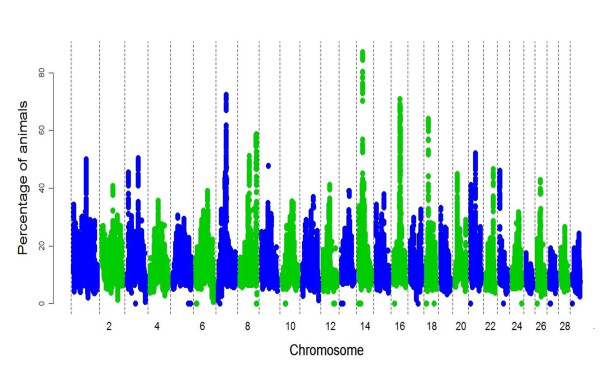
**Incidence of each single nucleotide polymorphism (SNP) in a Run of Homozygosity (ROH) in the HD panel population.** Particular genomic positions can be seen, on each of chromosomes 7, 8, 14, 16, 18 featured in ROH which were shared in over 55% of the sampled animals.

The number of ROH per chromosome was greatest for chromosome 1 (11,513 runs across all 867 animals with the HD panel) with on average, 12.39% of the chromosome consisting of a ROH. The number of ROH per chromosome tended to decrease with chromosome length (see Additional file 2). The fraction of chromosome residing in ROH was greatest on chromosomes 14 and 16, with 13.71% and 14.16%, respectively; these had the largest extent of overlapping ROH positions among individuals (see Additional file 3). In particular, 756 animals (87.2% of the dataset) had a ROH segment on chromosome 14 involving the same genomic region centred around the 25 Mb position that consisted of 28 SNPs and was 127.3 kb in length (Figure 2; Additional file 4). When interrogated using Haploview [13] the majority of SNPs in this region were in high LD with each other ( Additional file 5). Also, regions on each of chromosomes 7, 8, 14, 16, 18 featured in a ROH in over 55 % of the sampled animals (Figure 2). Chromosome 12 displayed the longest run of contiguous SNPs (197) that were uninvolved in any ROH within the sample population and interrogation in Haploview [13] revealed that this genomic region had corresponding low levels of LD between SNPs ( Additional file 6). The overlapping ROH regions on chromosomes 8 and 23 were found to be the most allelically similar with over 80% of these overlaps having a >95% allelic match. As this was only examined in the HD population it was difficult to discern a geographical pattern affecting this but the Hereford breed displayed the strongest effect on both chromosomes.

### Correlation between ROH and inbreeding coefficient

**Figure 3:**
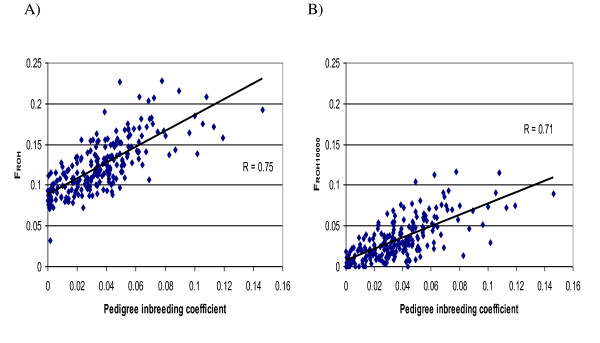
**Scatter plot of A) F**_**ROH**_**on pedigree inbreeding coefficient B) F**_**ROH10000**_**on pedigree Inbreeding coefficient.** F_ROH_ is the sum of all runs of homozygosity (ROH) per animal that are >0.5 Mb, F_ROH10000_ is the sum of all ROH per animal that are >10 Mb and the pedigree inbreeding coefficient is based on pedigree recorded data calculated by the Meuwissen and Lou algorithm (1992).

Cattle offer an opportunity to assess ROH as an indicator of inbreeding by comparing pedigree-based estimates of inbreeding using the same individuals. Figure 3 illustrates a scatter plot of either individual animal F_ROH_ or F_ROH10000_ (the sums of all ROH per animal that are >0.5 Mb and >10 Mb respectively) against the inbreeding coefficient based on pedigree recorded (McParland et al., 2007). Conservatively, we restricted our sample to the 230 animals that had complete generation equivalent values ≥6 of recorded pedigree ancestry. A clear linear relationship exists between the ROH and pedigree based estimates of inbreeding. The correlations between the pedigree inbreeding coefficient and individual sums of ROH were different from zero with values of r = 0.75 (P < 0.0001) for F_ROH_ and r = 0.71 (P < 0.0001) for F_ROH10000_ using the HD panel and r = 0.73 (P < 0.0001) for F_ROH_ and r = 0.70 (P < 0.0001) for F_ROH10000_ using the reduced HD panel. The intercept (0.088, s.e = 0.002) of the regression of F_ROH_ on the pedigree inbreeding coefficient was greater than zero suggesting that the pedigree-based inbreeding coefficient may underestimate the levels of ancestral genomic relatedness that may exist. The intercept (0.006; s.e = 0.002) of the regression of F_ROH10000_ on the pedigree inbreeding coefficient was also greater than zero, but to a lesser extent when the 10 Mb ROH length limit is applied; consistent with longer ROH resulting from more recent inbreeding, such as that recorded in these pedigrees.

### Validation of Bovine SNP50 density

**Figure 4:**
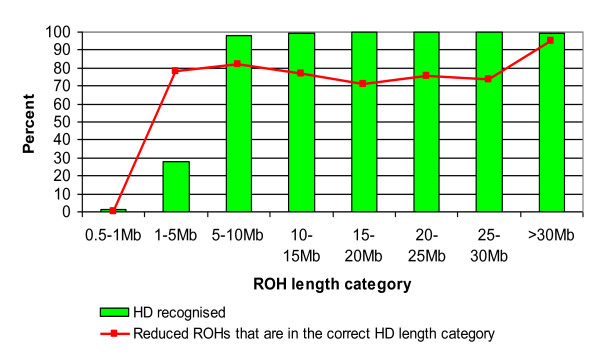
**The percentage of Runs of Homozygosity (ROH) in the HD panel that are recognised by the reduced density panel.** Also the percentage of the reduced panel ROH that are correctly identified into the correct ROH length category is also plotted. Greater than 70 % of the ROH identified using the reduced panel were placed in the correct length category. The reduced density failed to identify ROHs between 0.5-1 Mb in length due to the low SNP density.

The most common SNP density platform for cattle is that from 54,000 SNP genotyping arrays (Illumina BovineSNP50 beadchip); an important question is whether this panel can reliably infer ROH that have been detected with the greater accuracy provided by the HD array. To facilitate this, a reduced genotype dataset comprising 48,734 SNPs that were common to both the HD and Bovine50 Beadchip genotype panel on the 867 genotyped sires was used for comparison purposes. The same ROH criteria were applied to each dataset, where no more than two missing genotypes and one possible heterozygote were permitted per window, no restriction was placed on the number of SNPs in a ROH in either density and the maximum gap between two consecutive SNPs was kept at the default value of 1000 kb to account for the lesser genotype density in the reduced HD panel and to limit bias for comparison of ROH. There were in total 157,600 ROH identified using the HD panel, whereas only 19,078 ROH were identified in the same individuals using the reduced HD panel. However, the vast majority of ROH that the reduced panel failed to recognise were between 0.5-1 Mb in length (Figure 4). Also, only 27.7% of the 1–5 Mb ROH length category in the HD panel were identified by the reduced panel but almost all ROH in length categories of 5 Mb and above were identified (Figure 4). In the larger ROH length categories, ROH from the HD panel that were recognised by the reduced panel were correctly assigned >70% of the time to the correct length category where the majority of incorrectly assigned ROH were due to inflation of length in the reduced HD panel. When the reduced panel density ROH estimates were compared to pedigree inbreeding values using the same individuals the correlations obtained were very similar (0.73;P < 0.0001) using F_ROH_ and 0.70 (P < 0.0001) using F_ROH10000_ to those observed for the HD genotype panel.

### ROH in Bovine SNP50 genotypes across the 42 breeds

**Figure 5:**
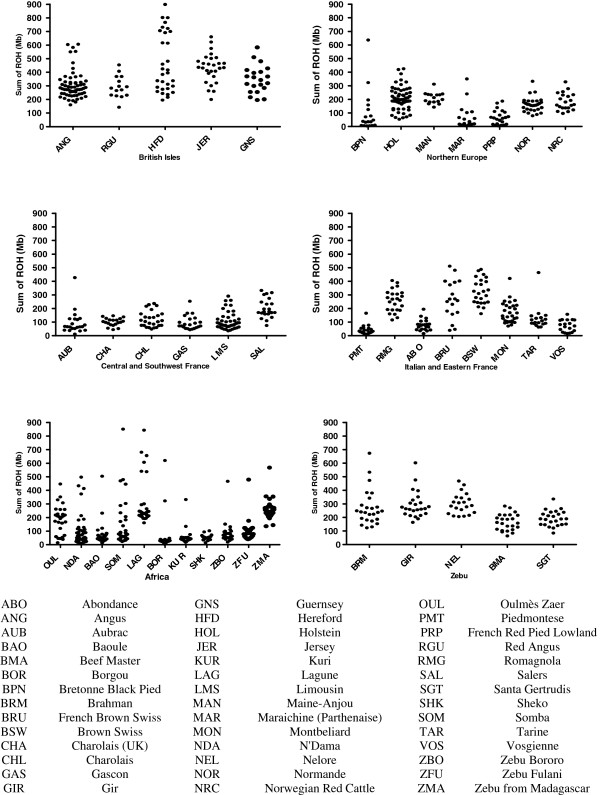
**Individual sum of total Run of Homozygosity (ROH) per Bovine SNP50 animal split into origin of breed.** The British Isles breeds had higher sums of ROH per animal in comparison to other geographic origins. Breeds originating in Africa have the most disparate range of sums a reflection of the current breeding practices.

The success of SNP50 validation exercise allows the testing of a large previously published data collection of wide provenance [7-9] for ROH distributions using 1 Mb as a ROH minimum. Figure 5 describes the total (Mb) per individual genome estimated to be under ROH in different breed groupings. There were clear differences among breeds in both the levels and variation of ROH frequency. Also, there were discernable patterns with respect to breed origin. The British Isles breed samples (Angus, Guernsey, Hereford and Jersey) clearly displayed the highest individual sum of ROH per animal. Mainland European breed samples showed varying ranges of ROH levels and zebu breeds had intermediate levels. African breeds had a tendency towards low ROH but the West African taurine breeds (Baoule, Lagune, N’Dama and Somba) showed high variability in individual sums of ROH within breed samples (Figure 5).

**Figure 6:**
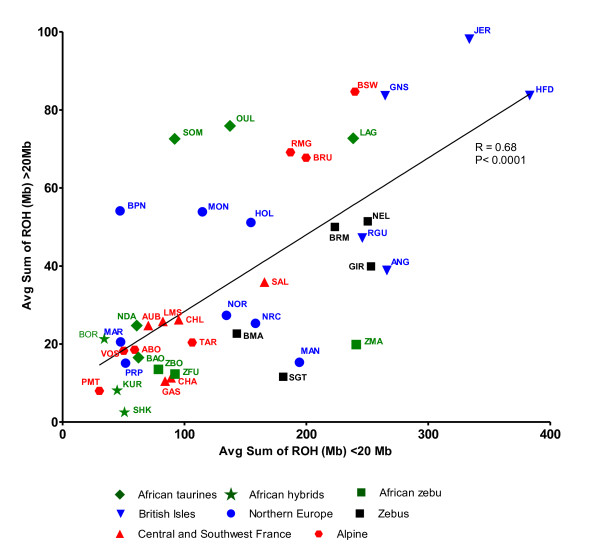
**Average sum of Runs of Homozygosity (ROH) per bovine SNP50 animal <20 Mb in length within breed VS average sum of ROH >20 Mb in length.** Breeds that originate from the same geographical area tend to cluster together with African *Bos taurus* breeds showing low levels for both measures, except for the Somba, Oulmès Zaer and Lagune breeds where elevated levels were clearly highly influenced by long ROH whereas the zebu breeds showed intermediate values of ROH but these were more strongly influenced by shorter ROH.

Long ROH arise as a result of recent inbreeding, while shorter ROH can indicate more distant ancestral effects such as breed founder effects. In order to discriminate between these effects the average sum of the genome under ROH of size >20 Mb against the average sum <20 Mb for each breed were compared (Figure 6). A correlation of r = 0.68 (P < 0.0001) existed between the two measures but the relative contributions of long and short ROH differed sharply among breeds and breed types. The African *Bos taurus* breeds showed low levels of ROH for both measures, except for the Somba, Oulmès Zaer and Lagune breeds where elevated levels were clearly highly influenced by long ROH and hence recent consanguinity. The two Channel Islands breeds also showed this trend. In contrast, the zebu breeds showed intermediate values of ROH but these were more strongly influenced by shorter ROH, perhaps indicating a founder effect rather than recent inbreeding.

## Discussion

Our findings show that ROH are frequent across all breeds and that certain ROH length categories can be used as an indication of consanguinity. They can also inform on breed population history as the effects of population bottlenecks, selection pressure and breeding management on the bovine genome may potentially leave an imprint on ROH length.

The bovine HD SNP assay allows an analysis of ROH at similar density to that employed to generate genomic signatures of endogamy that differ markedly among human populations [1-3]. Moreover, cattle allow a first comprehensive testing of the inference drawn from ROH through comparison with estimates of inbreeding from calculations based on the detailed pedigree data available for many breeds. The strong correlation between the pedigree inbreeding coefficient and sum of ROH of length > 0.5 kb suggests that, in the absence of an animal’s pedigree data, the extent of a genome under ROH may be used to infer aspects of recent population history even from relatively few samples, as previously suggested by McQuillan et al. (2008). However, 44% of the variance in ROH distribution remains unexplained by pedigree inbreeding and may partly reflect the limitations of ancestry recording in cattle where founder animals are generally, and often inaccurately, assumed to be unrelated (McParland et al., 2007). Additionally, the propensity for multiple megabase scale ancestral haplotypes in certain genome regions to persist even in outbred animals, perhaps due to localised low levels of recombination and high levels of LD may contribute [14]. Lastly, we note that pedigree relatedness gives an expected, not actual, proportion of genomic identity by descent among individuals and it might be anticipated that genotype-based estimates provide greater accuracy on relatedness [15].

Whereas HD SNP panels facilitate more accurate detection of ROH, the vast majority of cattle SNP genotype data, and emerging data in other livestock, is available at ~50,000 SNP density. It is therefore of interest as to whether these sparser genotypic data can reliably inform on ROH and inbreeding history. We found that HD ROH were not accurately identified in such a reduced panel if between 0.5-1 Mb in length. However, the SNP50 density genotypes were sufficient to recognise almost all ROH >5 Mb but they also has the potential to inflate ROH length. Importantly, ROH levels at the lower SNP density correlate equally well with the pedigree estimates of inbreeding. We conclude that this prevalent marker density is appropriate in identifying ROH.

We used three published [7-9] SNP50 genotype collections to examine patterns in ROH distribution and compare aspects of population history among a range of cattle breeds. The domestication process itself featured a limited sampling from the wild with a more recent bottleneck detectable 50–100 generations ago, presumably corresponding to breed formation [16]. However, this traditional breed formation is largely a European phenomenon and its absence is most apparent in the data from African cattle. These samples, including *B. taurus* breeds, humped *B. Indicus* breeds and indicine/taurine hybrids, tended toward low levels of ROH per genome, reflecting traditional management practices in Africa, characterised by less controlled mating [17].

An open village breeding system may also predispose to random consanguineous matings and many African breeds show outlying highly inbred individuals (Figure 5). The length distribution of ROH can help to distinguish different types of parental relatedness. Samples from human populations where cousin marriage is common show an excess of long ROH; whereas for example, Papuan and Melanesian human populations show an excess of shorter ROH, consistent with effects of reduced population and isolation rather than first degree relative unions (Kirin et al. 2010). Figure 6 compares the contributions of long (> 20 Mb) and short ROH to breed homozygosity in order to differentiate the effects of ancient and more recent relatedness among ancestors. Here, the three African taurine breeds (Oulmes zaer, Somba and Lagune) with higher homozygosity clearly show a strong influence of ROH of length greater than 20 Mb and hence of recent inbreeding. We note that Gautier et al. (2009) reported a high F_IS_ value, as well as extensive linkage disequilibrium within the Lagune breed.

Zebu-taurus hybridisation is also a dynamic and contemporary process within Africa [18-20]. This acts to increase genetic diversity and contributes to the interruption of stretches of homozygous genotypes within individuals. The effects of this process are evident in three hybrid breeds which show the lowest extent of ROH in African breeds. These include the Kuri; where previous work has shown a near 50:50 genetic admixture between surrounding zebu and themselves [21], and the Sheko breed, where the original taurine African *Y* chromosome is in danger of disappearing from this breed due to the use of zebu bulls [20].

Within European breeds, British breeds tended toward higher quantities of ROH, reflecting results of previous microsatellite research, where British Isles breeds had lower levels of observed heterozygosities and gene diversities in comparison to other Mediterranean and Northern European breeds analysed [22]. The Channel Island breeds showed strong influence from long ROH reflecting their unusually closed population histories due to strict importation restrictions on both the Jersey and Guernsey Islands implemented during the 1800 s [23].

The zebu breeds represented in this study have contrasting histories. The mainland African zebu breeds (Bororo and Fulani) which are products of ancient introductions from South Asia and are all hybrids to some extent, had much lower quantities of average ROH in comparison to the American zebu breeds analysed (Figure 6). Within the American and Madagascan zebu populations a stronger homozygosity signal, with a weighting toward smaller length ROH, suggests that these breeds were initially established by small founding populations but were not particularly affected by recent inbreeding (Figure 6). The initial introduction of the now prolific zebu animals in the Americas featured very limited numbers during the 19^th^ and 20^th^ century and Madagascan zebu were founded by ancient importations from Asia and East Africa which were probably limited in scope due to the isolation of the island [20,24].

The ascertainment bias [8] towards European *Bos taurus* breeds that is associated with the Bovine SNP50 genotyping chip, does not seem to invalidate the trends in ROH levels observed here, as ROH levels were in fact higher in those breeds with a higher number of polymorphic SNPs, as validated by Illumina ( Additional file 7). Also, the existence of long ROH (>20 Mb) for example in many of the less polymorphic African village breeds (Oulmes zaer, Somba and Lagune) are unlikely to be artefactual due to the vanishingly small probability of long contiguous homozygous SNPs occurring by chance. However, some bias may exist in the *Bos Indicus* ROH levels, as an over estimation of ROH amounts is possible due to low amount of polymorphic markers found in these breeds due to the design of the genotyping chip [8], as a result some caution must be taken when inferring ROH levels within these breeds. The bovine HD genotyping chip was designed from a more comprehensive range of breeds comprising several temperate and tropically adapted *Bos taurus, Bos indicus* and hybrid breeds and thus does not exhibit the same level of ascertainment bias [25].

The Hapmap population data also allow comparison with an alternative inference of past population size. Linkage disequilibrium may be used to infer past population size where higher r^2^ indicates lower effective population size with LD at longer genetic distances corresponding to younger time depths [26,27]. Interestingly, the Hapmap breed samples analysed here show a strikingly similar ranking in LD at distances of >200 kb to that which they show in average ROH [28].

Analyses of human ROH have previously established a correlation between extensive LD, locally low rates of recombination and high incidence of homozygous runs [2]. Intensive selection intensity in cattle has possibly acted to maintain long lengths of homozygous tracts. Previous work carried out in over 500 animals from 8 breeds noted that high levels of LD, particularly in the Holstein breed, existed on chromosomes 14 and 16, the two chromosomes with highest proportions of ROH in our study [14]. Conversely, chromosome 12 was found to have higher than average recombination rates and lower levels of LD (r^2^ <0.2) than the majority of chromosomes [14] and, interestingly, showed the highest proportion of SNPs uninvolved in a ROH within our sample population. The existence of recombination hotspots throughout the genome also can impact ROH, with multiple genomic regions that remained uninvolved in any ROH such as those on chromosomes 12 and 23 found to be well documented human and cattle recombination hotspots [29-31].

The existence of QTL in ROH have been well documented in human studies [5,6,32]. Here, several of the highly involved genomic regions located on chromosomes 7, 14, 16 and 18 (Figure 2) all potentially contain genes of importance in cattle with associations ranging from immunity through to carcass and dystocia related traits [33-35] when explored using three QTL databases available online ( http://genomes.sapac.edu.au/bovineqtl/index.html, a http://www.animalgenome.org/QTLdb/cattle.html, http://www.ncbi.nlm.nih.gov/). In particular, Chromosomes 9 and 5, which had the highest amount of long ROHs (>20 Mb), are well documented to contain QTL pertaining to milk fat yield and weight related traits respectively [36-39].

## Conclusion

ROH analyses quantifies a feature of genomic variation that may be used in inference of population history and to associate with important production and disease traits and perhaps signatures of selection. We show that ROH analysis in cattle provides a sufficient predictor of the pedigree inbreeding coefficient and the prevalent SNP50 genotyping array may be a sufficient tool to predict ROH in the genome. Patterns of ROH may be decomposed to highlight the effects of recent and more ancient ancestral relatedness and match known aspects of breed history in a sample set of wide provenance.

## Competing interests

The author(s) declares that there are no competing interests.

## Authors’ contributions

DCP participated in the design of the study, carried out the ROH identification, the data analysis and drafted the manuscript. DPB participated in the design of the study, the data analysis and helped to draft the manuscript. SMP calculated all pedigree based inbreeding coefficients. DGB conceived of the study, and participated in its design and coordination, and helped to draft the manuscript. All authors read and approved the final manuscript.

## Supplementary Material

Additional file 1 Details of animals genotyped with the Bovine SNP50 density panel. Click here for file

Additional file 2 The number of runs of homozygosity (ROH), in the HD panel population, per chromosome (bars) and the mean per animal percentage coverage of the chromosome covered by ROH graph. Click here for file

Additional file 3 The maximum amount of overlapping runs of homozygosity (ROH) per chromosome and the percentage of overlapping ROHs that are a >95% allelic match graph. Click here for file

Additional file 4 Proportion of appearances of each SNP on chromosome 14 in a ROH across dairy, beef and British animals graph. Click here for file

Additional file 5 Linkage disequilibrium plot using Haploview of the consensus overlap segment located on chromosome in 86% of the ROH. Click here for file

Additional file 6 Linkage disequilibrium plot using Haploview of SNPs on chromosome 12 that did not appear in a ROH. Click here for file

Additional file 7 Average total sum of Runs of Homozygosity (ROH) per animal within breed vs the number of validated polymorphic loci in the Bovine SNP50 genotyping beadchip graph. Click here for file
