# Supplementary material for: Runs of homozygosity and population history in cattle
Source: BMC Genet. 2012 Aug 14;13:70. doi: 10.1186/1471-2156-13-70 (PMC3502433; doi:10.1186/1471-2156-13-70)
Supplement: Additional file 1 — Details of animals genotyped with the Bovine SNP50 density panel. [file 1471-2156-13-70-S1.pdf]

Additional Table 1: Details of animals genotyped with the Bovine SNP50 density panel

Code	Name of breed	Classification of origin	Number of animals	Data Source
**ABO**	Abondance	Eastern France and Italy	22	Gautier, Laloë et al. 2010
**ANG**	Angus	British Isles	62	Matukumalli, Lawley et al. 2009
**AUB**	Aubrac	Central and Southwest France	22	Gautier, Laloë et al. 2010
**BPN**	Bretonne Black Pied	Northern Europe	18	Gautier, Laloë et al. 2010
**BRU**	French Brown Swiss	Eastern France and Italy	18	Gautier, Laloë et al. 2010
**BSW**	Brown Swiss	Eastern France and Italy	24	Matukumalli, Lawley et al. 2009
**CHA**	Charolais	Central and Southwest France	20	Gautier, Laloë et al. 2010
**CHL**	Charolais	Central and Southwest France	26	Matukumalli, Lawley et al. 2009
**GAS**	Gascon	Central and Southwest France	22	Gautier, Laloë et al. 2010
**GNS**	Guernsey	Central and Southwest France	21	Matukumalli, Lawley et al. 2009
**HFD**	Hereford	British Isles	32	Matukumalli, Lawley et al. 2009
**HOL**	Holstein	Northern Europe	64	Matukumalli, Lawley et al. 2009
**JER**	Jersey	British Isles	28	Matukumalli, Lawley et al. 2009
**LMS**	Limousin	Central and Southwest France	45	Matukumalli, Lawley et al. 2009
**MAN**	Maine-Anjou (Rouge des Près)	Northern Europe	16	Gautier, Laloë et al. 2010
**MAR**	Maraichine (Parthenaise)	Northern Europe	19	Gautier, Laloë et al. 2010
**MON**	Montbeliard	Eastern France and Italy	30	Gautier, Laloë et al. 2010
**NOR**	Normande	Northern Europe	30	Gautier, Laloë et al. 2010
**NRC**	Norwegian Red Cattle	Northern Europe	21	Matukumalli, Lawley et al. 2009
**PMT**	Piedmontese	Eastern France and Italy	24	Matukumalli, Lawley et al. 2009
**PRP**	French Red Pied	Northern Europe	22	Matukumalli,

	Lowland			Lawley et al. 2009
**RGU**	Red Angus	Northern Europe	15	Matukumalli, Lawley et al. 2009
**RMG**	Romagnola	Eastern France and Italy	24	Matukumalli, Lawley et al. 2009
**SAL**	Salers	Central and Southwest France	22	Gautier, Laloë et al. 2010
**TAR**	Tarine	Eastern France and Italy	18	Gautier, Laloë et al. 2010
**VOS**	Vosgienne	Eastern France and Italy	20	Gautier, Laloë et al. 2010
**BMA**	Beef Master	American and Indian zebus	24	Matukumalli, Lawley et al. 2009
**SGT**	Santa Gertrudis	American and Indian zebus	24	Matukumalli, Lawley et al. 2009
**OUL**	Oulmès Zaer	Africa	27	Gautier, Laloë et al. 2010
**BAO**	Baoule	Africa	29	Gautier, Laloë et al. 2010
**LAG**	Lagune	Africa	30	Gautier, Laloë et al. 2010
**NDA**	N'Dama	Africa	55	Gautier, Laloë et al. 2010
**SOM**	Somba	Africa	31	Gautier, Laloë et al. 2010
**BOR**	Borgou	Africa	30	Gautier, Laloë et al. 2010
**KUR**	Kuri	Africa	30	Gautier, Laloë et al. 2010
**ZBO**	Zebu Bororo	Africa	23	Gautier, Laloë et al. 20100
**ZFU**	Zebu Fulani	Africa	30	Gautier, Laloë et al. 2010
**ETH ZEB**	Ethiopian zebu	Africa	20	This study
**SHK**	Sheko	Africa	20	Matukumalli, Lawley et al. 2009
**ZMA**	Zebu from Madagascar	American and Indian zebus	30	Gautier, Laloë et al. 2010
**BRM**	Brahman	American and Indian zebus	25	Gautier, Laloë et al. 2010
**GIR**	Gir	American and Indian zebus	24	Gautier, Laloë et al. 2010
**NEL**	Nelore	American and Indian zebus	24	Gautier, Laloë et al. 2010
