# Supplementary figures and images for: Runs of homozygosity and population history in cattle
Source: BMC Genet. 2012 Aug 14;13:70. doi: 10.1186/1471-2156-13-70 (PMC3502433; doi:10.1186/1471-2156-13-70)

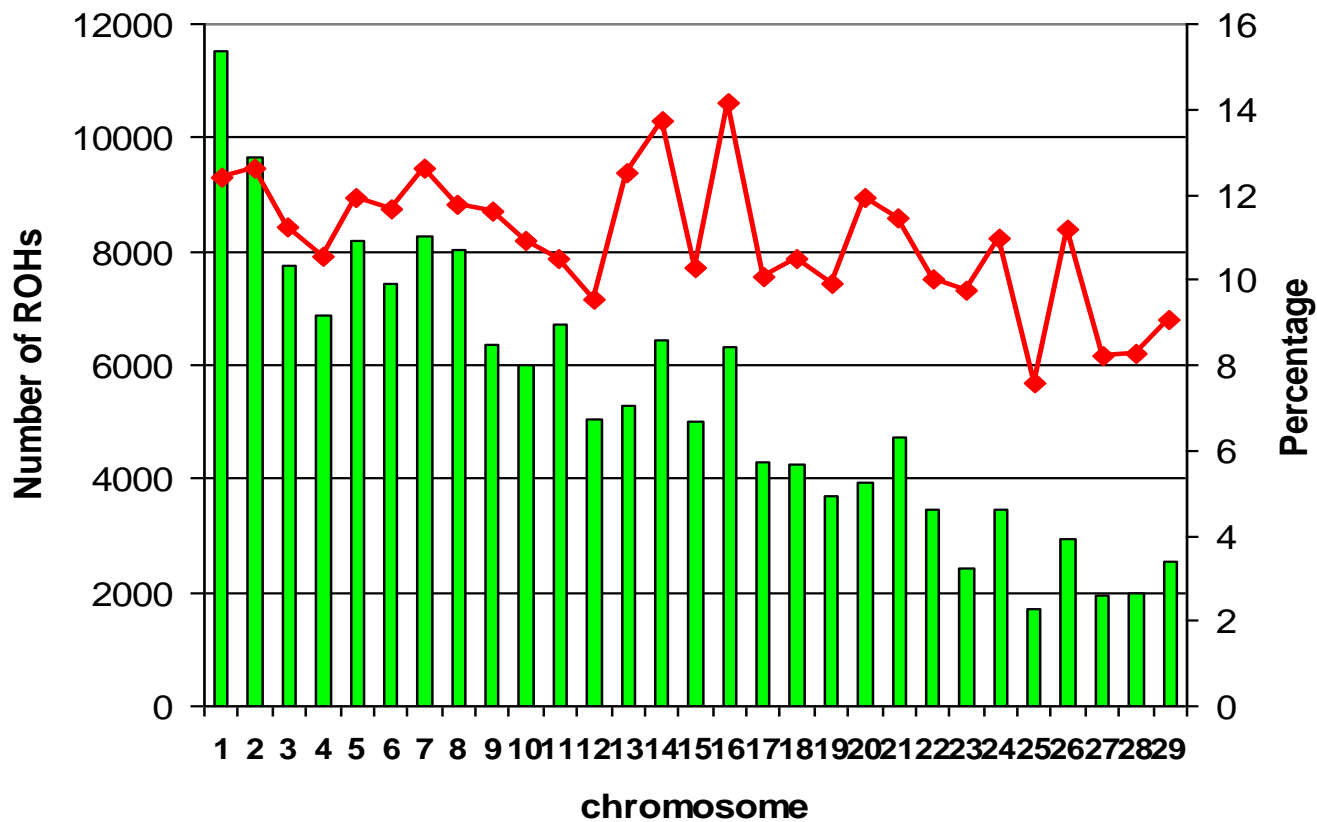

Supplement: Additional file 2 — The number of runs of homozygosity (ROH), in the HD panel population, per chromosome (bars) and the mean per animal percentage coverage of the chromosome covered by ROH graph. [file 1471-2156-13-70-S2.pdf]

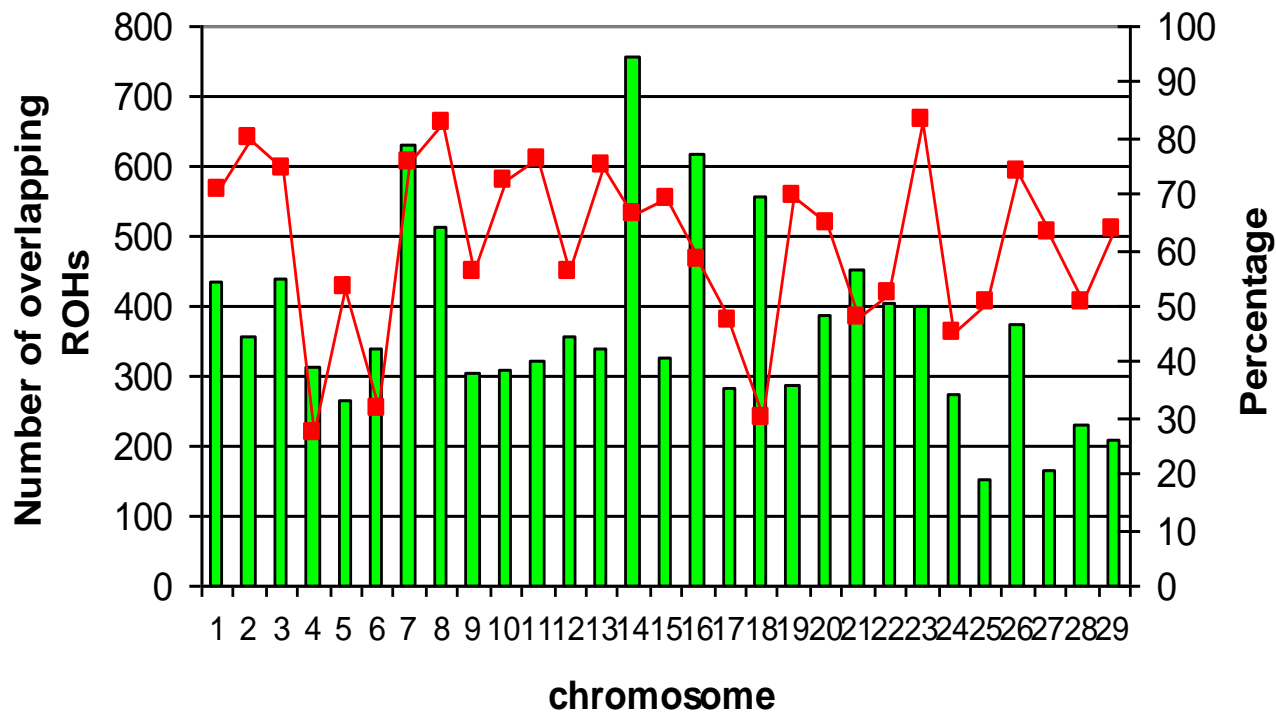

■ Number of overlaps —■ Percent allelic match

Supplement: Additional file 3 — The maximum amount of overlapping runs of homozygosity (ROH) per chromosome and the percentage of overlapping ROHs that are a >95% allelic match graph. [file 1471-2156-13-70-S3.pdf]

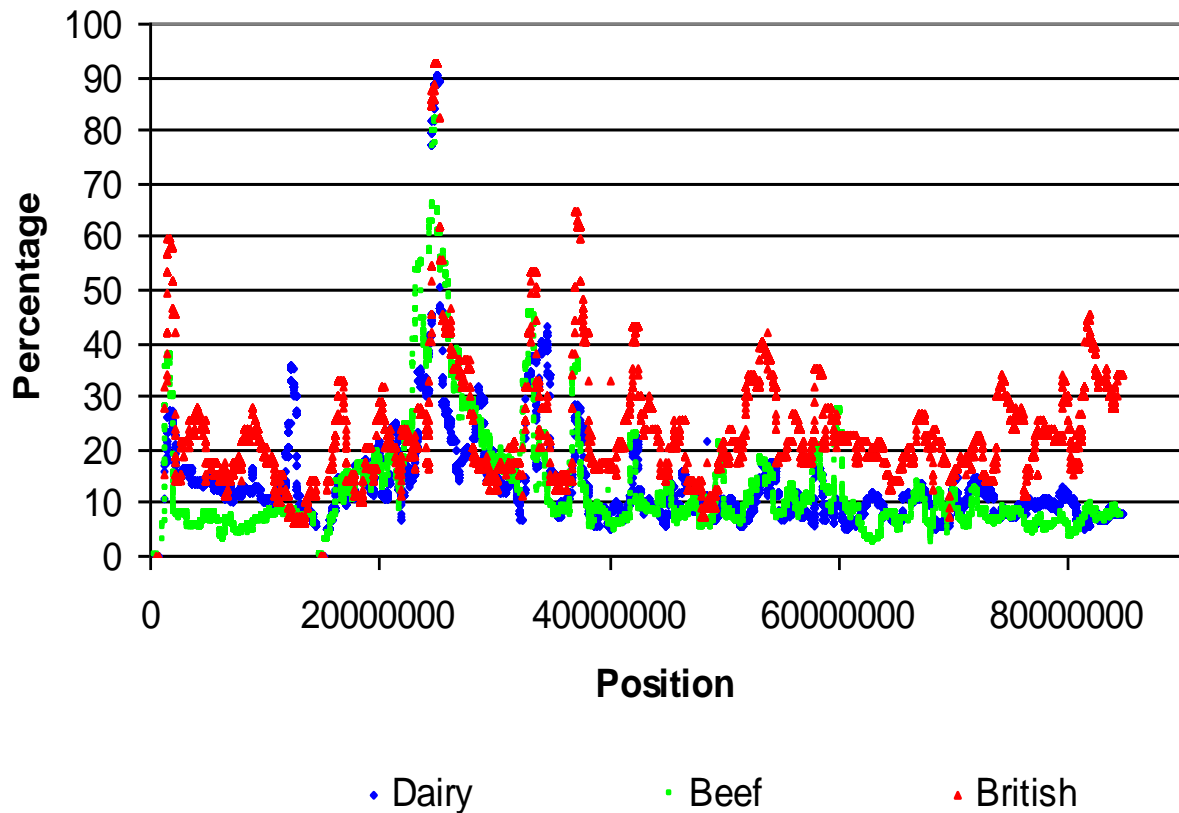

Supplement: Additional file 4 — Proportion of appearances of each SNP on chromosome 14 in a ROH across dairy, beef and British animals graph. [file 1471-2156-13-70-S4.pdf]

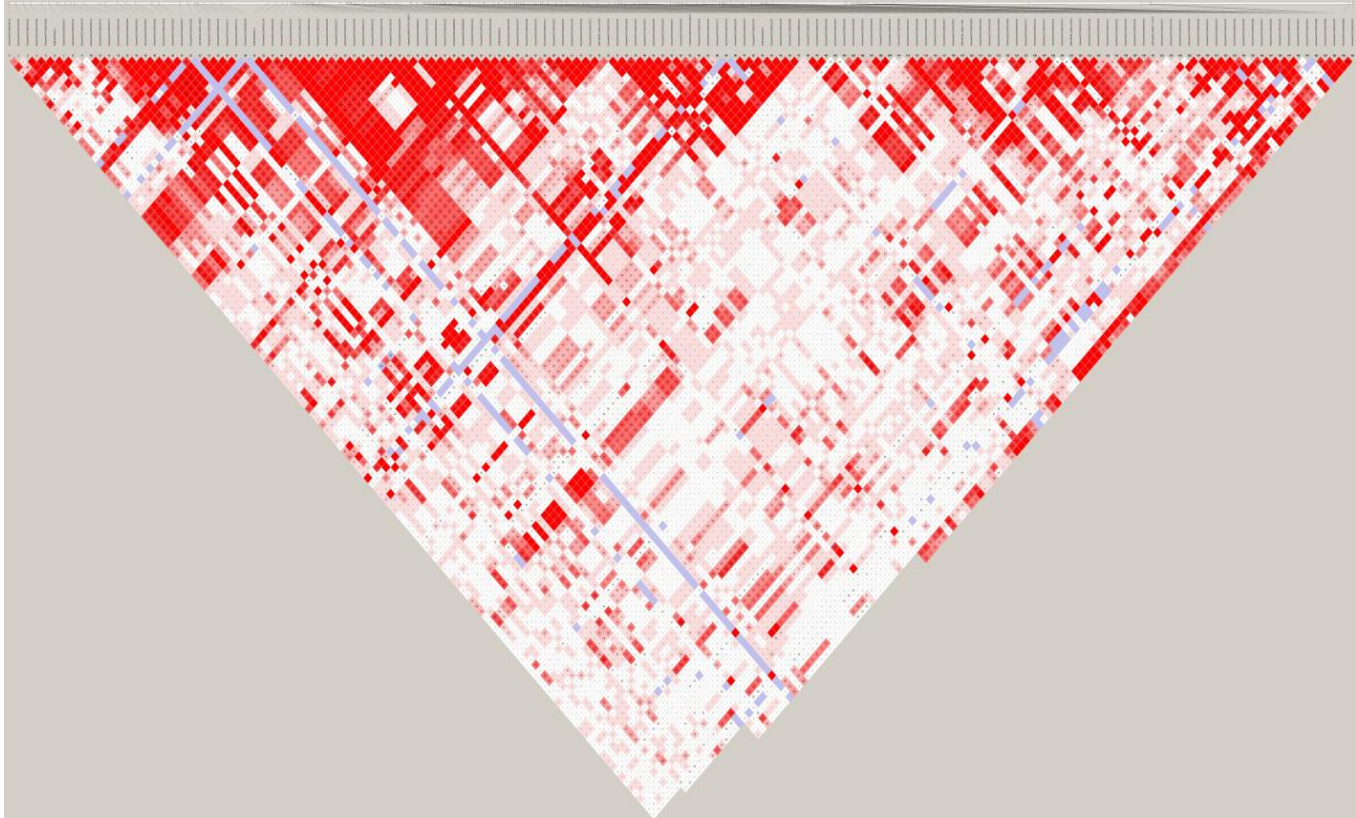

Supplement: Additional file 5 — Linkage disequilibrium plot using Haploview of the consensus overlap segment located on chromosome in 86% of the ROH. [file 1471-2156-13-70-S5.pdf]

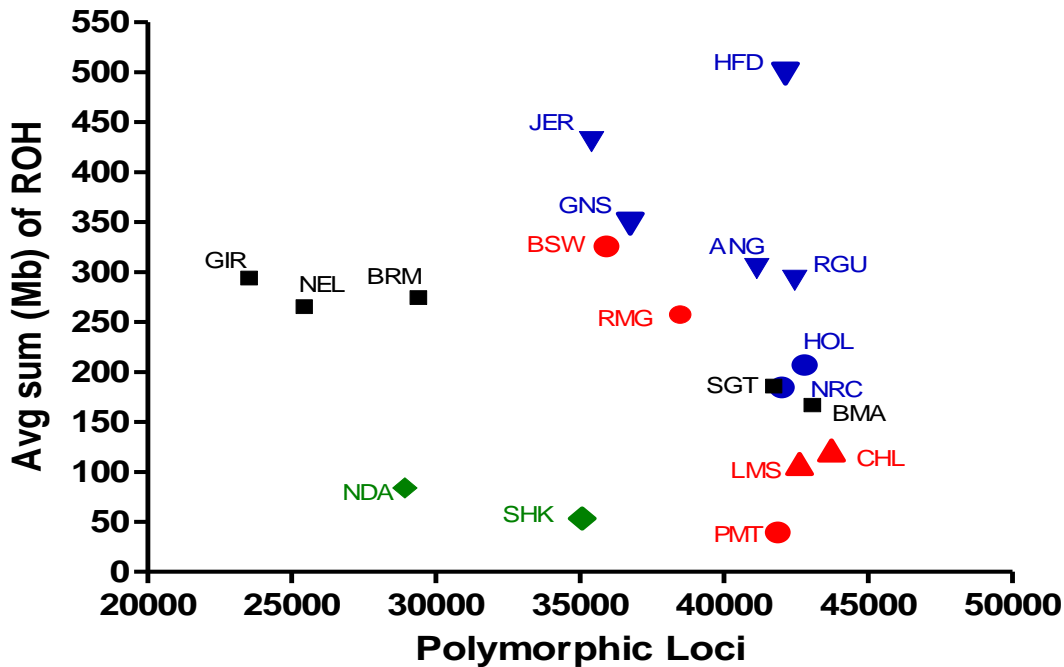

- |           |                                |
|-----------|--------------------------------|
| ◆ African | ▼ British Isles                |
| ● Alpine  | ● Northern Europe              |
| ■ Zebu    | ▲ Central and Southwest France |

Supplement: Additional file 7 — Average total sum of Runs of Homozygosity (ROH) per animal within breed vs the number of validated polymorphic loci in the Bovine SNP50 genotyping beadchip graph. [file 1471-2156-13-70-S7.pdf]
